# Genome Sequence of the Oleaginous Yeast Yarrowia lipolytica H222

**DOI:** 10.1128/MRA.01547-18

**Published:** 2019-01-24

**Authors:** Hugo Devillers, Cécile Neuvéglise

**Affiliations:** a Micalis Institute, INRA, AgroParisTech, Université Paris-Saclay, Jouy-en-Josas, France; University of California, Riverside

## Abstract

Here, we report the genome sequence of the oleaginous yeast Yarrowia lipolytica H222. *De novo* genome assembly shows three main chromosomal rearrangements compared to that of strain E150/CLIB122. This genomic resource will help integrate intraspecies diversity into synthetic biology projects that utilize Yarrowia as a biotechnological chassis for value-added chemical productions.

## Abstract (precis)

Here, we report the genome sequence of the oleaginous yeast Yarrowia lipolytica H222. *De novo* genome assembly shows three main chromosomal rearrangements compared to that of strain E150/CLIB122.

## ANNOUNCEMENT

The yeast Yarrowia lipolytica belongs to the “basal” lineages of the subphylum Saccharomycotina. Its oleaginous capacities in hydrophobic environments, the development of genetic tools, and a first available genome sequence in 2004 (1) have made it an interesting candidate for biotechnological applications for more than 30 years. Recent developments in synthetic biology and metabolic engineering have contributed to increasing interest in this yeast, which now emerges as a major host for chemical production (2). Here, we sequenced the genome of the German strain H222, which is one of the most utilized strains for biotechnological applications, such as production of organic acids (3–5).

Total genomic DNA of H222 cells grown in complete medium to the stationary phase was used to construct a shotgun 400-bp insert library (PE) and a mate pair 8-kb insert library (MP). Both libraries were sequenced in paired-end (2 × 100 bp) using the Illumina HiSeq 2000 platform with chemistry v3 (PE) and v2 (MP), resulting in a raw sequencing depth of 275× (28,182,153 reads) and 47× (4,798,539 reads) for PE and MP, respectively. Sequencing reads were cleaned using Trimmomatic v0.32 (6) and Cutadapt (7) with the options ILLUMINACLIP:<adaptors.fasta>:2:15:5 LEADING:5 TRAILING:5 SLIDINGWINDOW:5:20 MINLEN:36 and –error-rate = 0.2, respectively. Note that Cutadapt was used only for 5′ adaptor clipping. After trimming, 26,533,605 PE reads (255×) and 1,325,112 MP reads (13×) were used for *de novo* genome assembly using SOAPdenovo2 v2.04 (8), with a k-mer value of 77, as estimated with kmergenie version 1.67 (9). Gap closure was performed using GapCloser v1.12 (8). The final assembly comprised 17 scaffolds larger than 5 kb (*N*_50_ of 3.9 Mb, obtained with three scaffolds) for a cumulative length of 20,519,037 bp. A single scaffold of 48,435 bp corresponded to mitochondrial DNA. The remaining 16 scaffolds were suitable for automatic annotation using Rapid Annotation Transfer Tool (RATT) (10) with the Y. lipolytica E150 genome sequence as a reference (genome sequence available at http://gryc.inra.fr). Manual curation was performed with transcriptome sequencing (RNA-Seq) reads (BioProject accession number PRJEB29941) mapped to the assembly with TopHat2 (11). In total, 6,490 protein-coding genes were predicted, including 6,415 coding sequences (CDS) and 128 pseudogenes. A set of 510 nuclear tRNA genes were identified using tRNAscan-SE v1.3.1 (12). Transposable elements (TE) were identified by a BLAST search using different TE families from Y. lipolytica (13–18). A total of 88 solo long terminal repeats (LTR), mainly from Tyl5 (19), and 108 intact or remnant TE were annotated, including 97 copies of Ylli (13).

A draft genome sequence of H222 is already available (assembly ASM305430v1, submitted by Patrice Lubuta’s laboratory). However, the assembly is probably incorrect, since it is completely colinear to the genome of E150/CLIB122 (1), even though large differences have been observed in their karyotypes (20). In our assembly, we found two major reciprocal translocations compared to E150 chromosomes, involving scaffolds H222S03/S06 and H222S04/S08, and a large inversion of 300 kb in H222S01 compared to chromosome YALI0E of E150. This explains the observed chromosome size differences and shows that chromosomal rearrangements occurred in Y. lipolytica. Consequently, *de novo* assembly should be preferred over reference-assisted scaffolding for this species.

### Data availability.

The draft genome sequence of Yarrowia lipolytica strain H222 has been deposited in DDBJ/ENA/GenBank under the accession number GCA_900537225. The version described in this paper is the first version. The accession number for the project is PRJEB28424, and for the reads, ERR2767096 (PE), ERR2767094 (MP), and ERR2767095 (MP). The accession numbers of the 17 scaffolds are UTQH01000001 to UTQH01000017. Genome sequences and annotations are also available at the GRYC server (http://gryc.inra.fr).
